# Demography, heritability and genetic correlation of feline hip dysplasia and response to selection in a health screening programme

**DOI:** 10.1038/s41598-019-53904-w

**Published:** 2019-11-20

**Authors:** Matthew Low, Per Eksell, Kjell Högström, Ulrika Olsson, Lars Audell, Åsa Ohlsson

**Affiliations:** 1 Department of Ecology, Swedish University of Agricultural Sciences, Uppsala, Sweden; 2 XL Vet AB, Postvägen 7, Örbyhus, Sweden; 3 PawPeds, Gagnef, Sweden; 4 Gudby Djurklinik, Gudby gård, Upplands Väsby, Sweden; 5 Institute for Genetic Disease Control, Warner, NH USA; 6 Department of Animal Breeding and Genetic Health, Swedish University of Agricultural Sciences, Uppsala, Sweden

**Keywords:** Animal breeding, Diseases

## Abstract

Feline hip dysplasia (FHD) is a debilitating condition affecting the hip joints of millions of domestic cats worldwide. Despite this, little is known about FHD except that it is relatively common in the large breed Maine Coon. We used 20 years of data from 5038 pedigree-registered Maine Coon cats in a radiographic health screening programme for FHD to determine, for the first time, its heritability, genetic correlation to body mass and response to selection. FHD prevalence was 37.4%, with no sex predilection; however, FHD severity increased with age and body mass. Heritability of the radiographic categories used to classify FHD severity was 0.36 (95%CI: 0.30–0.43). The severity of FHD symptoms was also genetically correlated with body mass (0.285), suggesting that selection for a large body type in this breed concurrently selects for FHD. Support for this was found by following generational responses to selective breeding against FHD. Not only did selective breeding successfully reduce the severity of FHD symptoms in descendants, but these cats were also smaller than their ancestors (−33g per generation). This study highlights the value of breeding programmes against FHD and cautions against breed standards that actively encourage large bodied cats.

## Introduction

Hip dysplasia is a degenerative condition affecting the coxofemoral joint, where abnormal anatomical development and weight-bearing forces combine to create ongoing degenerative joint disease^1,2^. Because of its debilitating impacts on individual animals, considerable research effort has focussed on the disease’s genetic and environmental risk factors in order to mitigate these risks. Much of this work has been done in dogs because hip dysplasia is relatively common in larger dog breeds^2–5^; thus in dogs we know much about its breed predisposition, heritability, genetic regulation, environmental risk factors, symptoms and radiographic presentation, welfare implications and response to selection in health screening programmes^1–4,6–9^. Selective breeding against canine hip dysplasia through breed-specific programmes has become the mainstay of managing the prevalence of the disease for over 50 years^2,3,6,10^. However unlike dogs, hip dysplasia in cats has been largely overlooked and underreported^11–13^, with very little known about its demography (but see^14^), and almost nothing known about risk factors, heritability or potential response to selection^11,13,14^.

Current knowledge about feline hip dysplasia (FHD) comes largely from individual case reports in the veterinary literature^15–17^, and four studies reporting its breed-specific prevalence^11,14,18,19^. While there is evidence that FHD may be more common in some breeds (e.g. Devon Rex, Himalayan & Persian^11,19^), these differences are often difficult to quantify because of the small sample sizes reported (e.g. the 78 cats examined in^19^ are divided among 9 breeds). Despite this, there are clear indications that domestic shorthairs have a lower prevalence of FHD (10.4% from 899 cats^11,18,19^) compared to Maine Coons (24.9% from 2548 cats^14^). Because of these likely breed associations, FHD is expected to have a genetic basis similar to the polygenic condition in dogs^11,13,20,21^. Thus it is assumed that FHD can be similarly managed, with selective breeding and culling being used to limit its impact in specific populations. However, while these assumptions are reasonable, the heritability of FHD and its subsequent response to selection are still unknown; issues that have significant implications for our understanding and management of FHD^6,11,21^.

**Figure 1:**
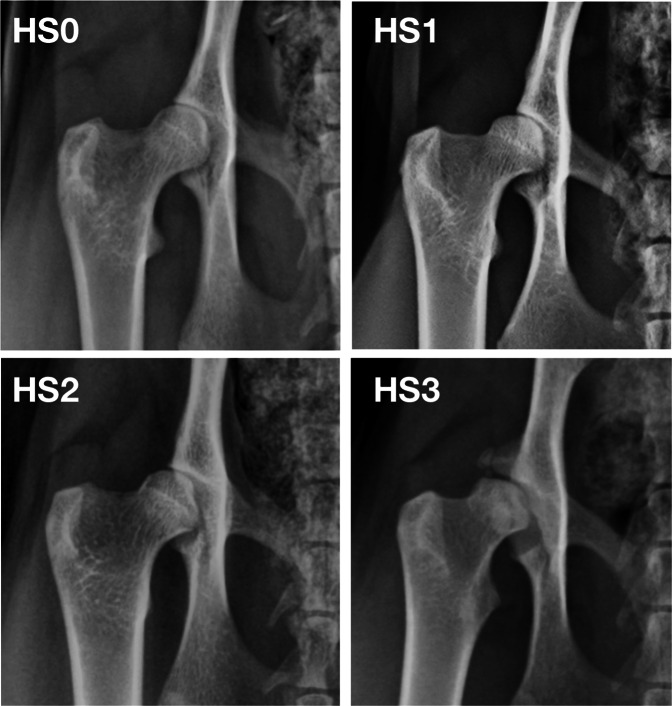
Examples of hip radiographs (right coxofemoral joint) taken from the PawPeds database showing examples of the range of possible hip score grades in the programme: (top left) hip score = 0 (HS0) normal hip with no evidence of FHD; (top right) hip score = 1 (HS1) hip with acetabulum covering < 50% of the femoral head; (bottom left) hip score = 2 (HS2) moderate radiographic signs associated with FHD including shallow acetabulum and deformation of the femoral head with some evidence of new bone formation around the joint; (bottom right) hip score = 3 (HS3) severe radiographic signs associated with FHD with very poor joint congruency, deformation of the femoral head and major changes associated with new bone formation around the joint.

Heritability is the proportion of phenotypic variation arising from additive genetic effects, and is critical for estimating the expected response to selection^22^. To determine heritability of a phenotypic trait, in this case FHD, we need many individuals of known genetic relationship to one another whose FHD symptoms can be compared. Currently in cats, such an analysis is only feasible in the Maine Coon. Two international registries collect data on FHD in the Maine Coon: the Orthopedic Foundation for Animals (OFA) whose demographics are described in Loder & Todhunter^14^ and the Swedish-based PawPeds programme (https://pawpeds.com). In both programmes, FHD is classified into ranked scores based on the severity of radiographic signs (Fig. 1). The PawPeds programme also bases breeding recommendations on these FHD scores, with breeders strongly encouraged to selectively breed for a lower incidence of FHD. Organised selective breeding against disease traits is much less common in cats compared to dogs; thus, PawPeds data offer an unusual opportunity to study the success of breeding programmes in domestic cats. These factors, combined with the Maine Coon pedigree registry also managed by PawPeds, allows not only an assessment of FHD demographics (e.g.^14^), but also an estimate of its heritability and response to selective breeding.

We analysed data collected by the PawPeds FHD Maine Coon health programme and linked these data to the Maine Coon pedigree registry. This allowed us to specifically examine: (1) demographics of hip dysplasia from 5038 Maine Coon cats and its relationship to probable risk factors (i.e. sex, age, body mass and year of the health programme), (2) response to selective breeding, by looking at the relationship between FHD scores and the number of generations within the selective breeding programme, and (3) the heritability of FHD, by using the linked pedigree to calculate the ratio of additive genetic variance to phenotypic variance. The Maine Coon is a large cat breed, and this in combination with evidence that hip dysplasia in dogs is associated with large dog breeds^3,5^, suggests some linkage between genes for body size and hip dysplasia risk. Thus, we also examined: (4) the genetic correlation between FHD scores and body mass to assess how selection for size in this large breed may concurrently select for FHD. By extension, we also examined whether selection against FHD in the health programme concurrently selected for a smaller body mass in subsequent generations [because of this genetic correlation]. These questions are not only invaluable for greatly expanding our currently limited knowledge of FHD, but also for validating the effectiveness of current and future breeding programmes. Information regarding genetic correlations between FHD and body mass will have significant implications for the breed standard and the sorts of traits breeders should be selecting for if they want to limit FHD in this (and other) cat breed(s).

## Results

### Prevalence and factors related to HD expression

**Figure 2:**
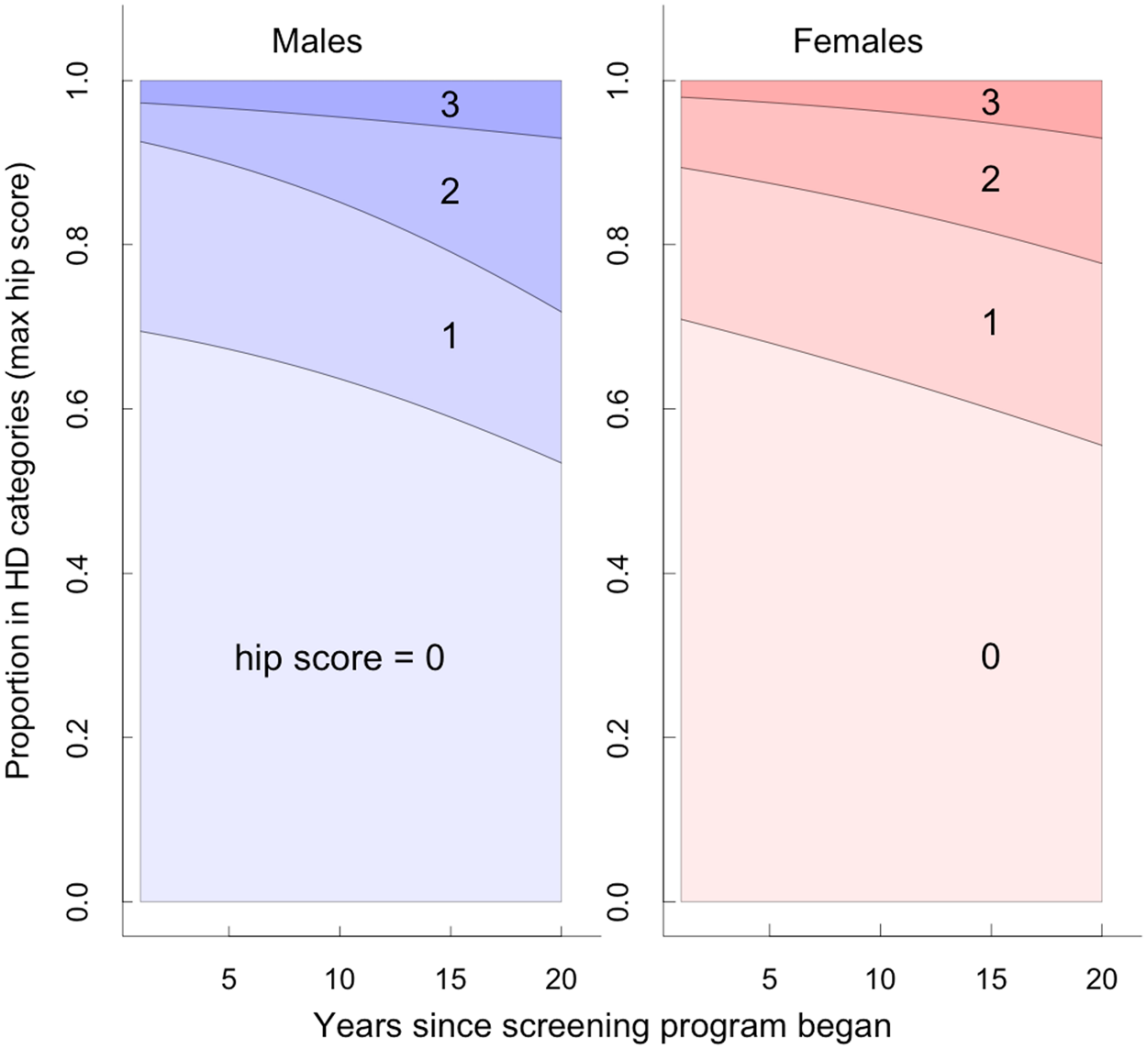
Cross-sectional data of 5038 individual FHD assessments showing the estimated proportion of cats in the different hip score categories for hip dysplasia (HD) based on radiographic evaluation (0 = no sign, 1 = mild, 2 = moderate, 3 = severe; Fig. 1). Cats were categorised based on the hip with the highest (max) HD score. Estimates are from sex-specific multinomial models relative to the years since the health screening and selective breeding programme was initiated in 2000.

**Figure 3:**
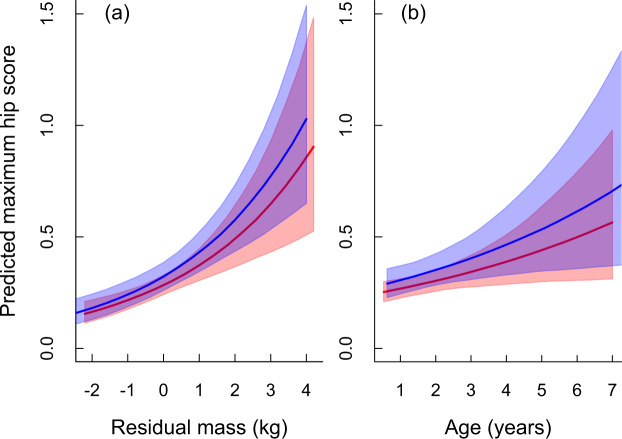
Estimated relationship between hip scores based on radiographic signs of FHD and (**a**) residual body mass, and (**b**) age. In both panels the red and blue lines show the mean sex-specific predictions (females vs. males respectively) with the associated shaded areas the 95% credible intervals of the prediction. Cats were categorised based on the hip with the highest HD score (maximum). Predictions are derived from the model in Appendix S1a.

More than one third of all Maine Coon cats surveyed showed some radiographic signs of hip dysplasia (37.4%), with the proportion of cats in each HD category being approximately the same for males and females (i.e. hip score 1 = 22%, score 2 = 12% and score 3 = 4%; Table 1). For cats with radiographic signs of FHD, 36.9% had lesions in only one hip and 63.1% in both hips; males and females were similar in proportion and range of severity (Supplementary Table S1; Fig. S1). During the 20 years of health monitoring, the proportion of cats without radiographic signs of FHD declined marginally (Fig. 2), with the proportion of cats in the most severe FHD categories (2 & 3) increasing (Fig. 2; Supplementary Fig. S2; Table S2). Residual body mass and age were related to the severity of FHD expression, with heavier and older cats having higher than average hip scores compared to lighter or younger cats (Fig. 3; Supplementary Table S3).

**Table 1:** Hip scores from FHD evaluations for 5038 Maine Coon cats from 2000–2019 in the PawPeds hip dysplasia health monitoring programme based in Sweden.

Hip score	Males (n = 1751)			Females (n = 3287)		
	left	right	maximum	left	right	maximum
0	1217	1197	1085 (0.620)	2309	2279	2068 (0.629)
1	312	331	372 (0.212)	605	630	723 (0.220)
2	165	157	217 (0.124)	287	282	370 (0.113)
3	57	66	77 (0.044)	86	96	126 (0.038)

Hip scores range from 0 (no dysplasia) to 3 (severe dysplasia) and are assessed on both the left and right hips for each animal. Here these data are displayed for male and female cats, as well as the data distribution for each individual’s maximum hip score (e.g. if an individual scored left hip = 0, and right hip = 2, then their maximum = 2). Raw count data are shown; in parentheses, the proportion of the surveyed population with each maximum hip score is also given for ease of comparison between groups and sexes. In our GLMM and *h*^2^ analyses we use individual maximum hip scores as the main response variable. See Table S2 for additional information about laterality of FHD.

### Response to selective breeding

**Figure 4:**
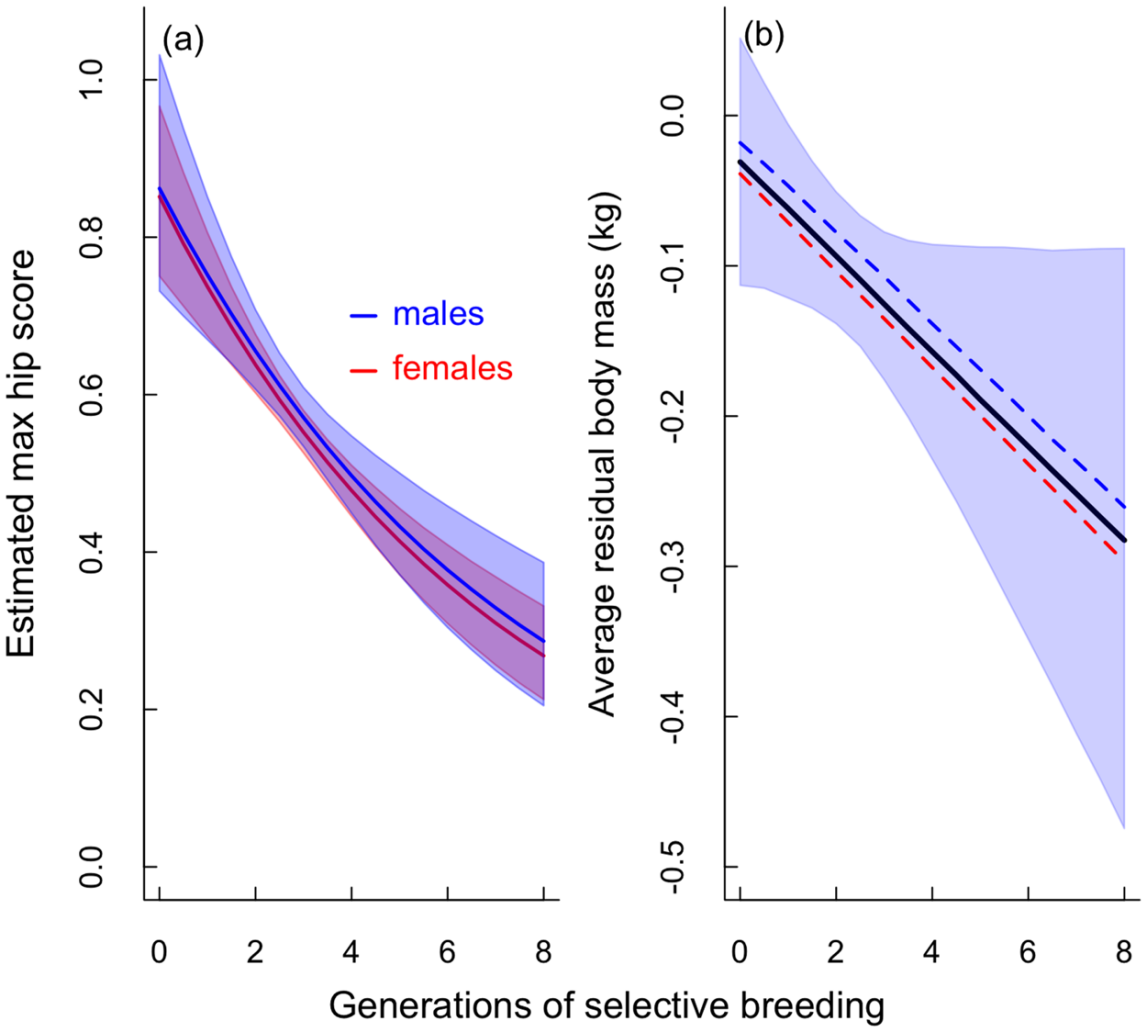
Estimated declines in (**a**) maximum hip score, and (**b**) residual body mass (in kg at the mean age of 520 days) relative to the number of generations of selective breeding against FHD. In (**a**) the red and blue lines show the mean sex-specific predictions (females vs. males respectively), with the associated shaded areas the 95% credible intervals of the prediction. In (**b**) the black line is the global mean prediction of residual body mass, with the shaded area the 95% credible intervals. The red and blue dashed lines show the sex-specific mean estimates (females and males respectively). Predictions are derived from the models in Appendix S1b,e.

Despite the cross-sectional data showing no reduction in the prevalence or severity of FHD in Maine Coons over time (Fig. 2), there was strong evidence from the multi-generational longitudinal data that selective breeding reduced FHD scores. Here, the predicted average hip score approximately halved after five generations of selective breeding (Fig. 4a); after eight generations the predicted maximum hip score per individual had declined to 0.268 (95% CI: 0.21–0.33) for females and 0.286 (0.21–0.39) for males (from a starting value at generation 1 of 0.85 (0.75–0.96) & 0.86 (0.73–1.03) respectively; Fig. 4a). This effect of culling animals with high FHD scores and only breeding from animals with normal hips or low FHD scores, thus resulted in a 68% (95%CI: 62–73%) reduction in the mean value of the hip score for females and a 66% (60–72%) reduction for males (assuming hip scores follow an approximately linear ordinal scale) between generations 0 and 8 (see Supplementary Table S3 for model coefficient estimates).

### Heritability and genetic correlations of HD in Maine Coons

The response to selective breeding was further supported by the quantitative genetic analyses demonstrating moderate heritability of HD scores (*h*^2^ = 0.36 at the observed data scale; Table 2). Genetic correlation analyses using the bivariate response traits of residual body mass and radiographic HD scores showed that these factors were moderately positively correlated (*r*_*G*_ = 0.285; Table 2). Thus, individuals with a genetic predisposition to be larger than average also had higher than average FHD scores because of some genetic linkage between these traits.

**Table 2:** Quantitative genetic estimates of heritability and the genetic correlation between heritable traits for Maine Coons derived from models in Appendix S1c,d.

	Estimate	95% CI range
**Heritability** ***h***^2^		
max hip score (latent scale)	0.51	0.42–0.60
max hip score (data scale)	0.36	0.30–0.43
residual body mass	0.57	0.37–0.77
**Genetic correlation** ***r***_***G***_		
max hip: residual body mass	0.285	0.044–0.424
left hip: right hip	0.983	0.975–0.995
left/right hip: max hip	0.996	0.995–0.998

Heritability is estimated for the maximum hip score and residual body mass. Because hip score data are ordinal, raw heritability estimates are based on the latent statistical variable and must be converted to the observed data scale to account for variance in the GLMM link function^31^; we present both estimates here. Estimates are means of the posterior distribution, with the associated 95% credible interval.

To confirm this finding and to examine a potential consequence of this genetic correlation, we considered whether selection against FHD within the health screening programme had an incidental effect on body mass. Here we expected that residual body mass should decline as a consequence of HD being selected against. We regressed residual body mass against the number of generations of selective breeding and found clear evidence for a reduction in body size (mean ~0.25 kg after 8 generations) when breeders select against FHD (Fig. 4b; Supplementary Table S3).

## Discussion

Our study confirms the high prevalence of FHD in the Maine Coon (37.4%; cf. 24.9% in^14^) and provides additional justification for the screening and selective breeding programmes that have developed around this cat breed. However it is naive to think that FHD is a condition largely restricted to the Maine Coon. The Maine Coon was the focus of this study simply because it is the only breed with data routinely collected on FHD. Previous studies show that other cat breeds likely suffer from this condition (e.g. Devon Rex [40%], Abyssinian [30%], Himalayan [25%], Persian [15%]^11,19^); however these are based on very small sample sizes (between 5 and 25 cats) because organisations associated with these breeds have not made specific recommendations to their members to screen for FHD. The only other type of cat with sufficient sampling to provide a good estimate of FHD prevalence is the domestic shorthair, where a combination of studies provides an estimate of 10.4% from a total of 899 cats^11,18,19^. This clearly suggests that FHD has a similar prevalence to canine HD (overall prevalence = 15.6%; breed ranges 0–77%)^5^. These findings are in stark contrast to Hayes *et al*.^20^ who estimated FHD prevalence to be extremely low (at 1/180th the prevalence of canine HD, based on 14 cases in 270,000 hospital visits), but neglected to consider that many of these patients would have had FHD but were not evaluated for it because cats do not show the same obvious clinical symptoms as dogs^12,13^. Thus, it is clear there has been a severe underestimation of FHD prevalence in the general cat population.

As with previous studies we found no association between gender and FHD prevalence or severity (Table 1). Laterality of FHD (when present) was relatively common with 37% of females and males presenting with an FHD score of >0 in only one hip (c.f. 45% and 43% in^14^). As has been previously noted, when bilateral HD occurs the severity of the condition is generally higher than when only one hip is involved (Supplementary Table S1)^5,14^. The explanation for this relationship between laterality and HD severity can be likened to sampling error from a probability distribution; cats with a genetic predisposition for mild FHD are more likely to have a normal hip by chance (and hence unilateral HD) than those with a genetic predisposition for severe FHD (hence they get bilateral HD more often; see Fig. S3 for an expanded explanation). Perhaps not surprisingly with a degenerative joint condition, our results show that FHD severity was related to age and the residual body mass of the individual (i.e. the deviation from the expected body size given the animal’s sex and age). Thus, older and heavier cats tended to have more severe FHD than younger and lighter cats. These relationships are important to consider when examining general FHD patterns in data, as non-uniform distributions of age or body mass within a dataset could lead to spurious conclusions if not accounted for.

Any effectiveness of the PawPeds health programme was not apparent when the raw cross-sectional data were initially examined. Over the course of the programme we expected the proportion of cats without FHD symptoms to increase because FHD was being selected against. Contrary to this, the proportion of cats in the moderate to severe FHD categories increased at the expense of those in the mild to no symptom categories (Fig. 2). At first glance this seemed to indicate the programme was not working. However, interpreting the cross-sectional data in this way depended on some very strong assumptions about the state of new cats entering the programme in later years being the same as those in the early years (age, body mass, severity of symptoms). By analysing longitudinal data that followed generations through the programme, we could see that selective breeding had a dramatic effect on the severity of hip scores. Thus, the lack of patterns in the expected direction in the cross sectional data likely arose from changes in the types of animals being submitted to the programme, with there being some evidence in the data that residual body mass increased during the life of the programme.

Our estimates of heritability (*h*^2^) of FHD were within the range commonly reported for HD in dogs (0.1–0.7)^1–5^, suggesting a similar polygenic aetiology and an expected response to selective breeding. But it is important to consider exactly what is meant by *h*^2^ of FHD and why *h*^2^ estimates will likely vary between this study and future studies. Heritability is the proportion of phenotypic variance in our measure of FHD that is explained by additive genetic factors. Thus, one needs to consider the amount of variation in the population and the relative contribution of genetic versus non-genetic effects. This means that variation arising from different gene pools or differences in the general rearing conditions or nutrition of the animals being evaluated may significantly impact the *h*^2^ estimates, with these potentially differing because of spatio-temporal or sampling issues. But potentially more importantly is the way FHD is measured or categorised in the first place. Heritability is calculated from the phenotypic variance, with the phenotype in this case not being FHD itself, but how we measure FHD. PawPeds uses a 4-category ordinal ranking to define FHD severity and grades the cat based on the highest (worst) hip score, while the Orthopedic Foundation for Animals (OFA) registry uses a 7-category grading system and grades the cat based on an average of the two hip scores^14^. While these ways of categorising FHD are likely to be very closely aligned and thus should reveal similar *h*^2^ estimates, any differences will probably say more about the method used to categorise FHD rather than the underlying gene pool. Thus, where future work focuses on the relative advantages of different methods for diagnosing or categorising FHD (e.g. rank scores versus Norberg angles, laxity scores or subluxation indices)^19,23^, it should be kept in mind that these diagnostic methods and their phenotypic variance may influence *h*_2_ estimates, with this, in turn influencing the effectiveness of selective breeding programmes based on these FHD scores^6^. In addition, *h*^2^ estimates could be influenced through observational error depending on the grading system used in the selection programme. Here we used a single observer when categorising FHD, whereas other programmes use multiple observers^14^. Single observer programmes potentially limit between-observer variation^3,24^ and hence produce higher and more accurate *h*^2^ estimates; however, they run the risk of introducing systematic bias during the course of the programme if the observer subtly changes the magnitude of their assessments over time. The degree to which these observer errors impact on the effectiveness of FHD screening programmes is unknown, but could potentially be investigated using the PawPeds and Orthopedic Foundation for Animals data. Currently, our study shows that the method used by PawPeds provides a measure with a moderate level of heritability for selection to act on and with direct evidence of declining FHD scores in response to selection. Thus there is no reason to change the way FHD is classified in this programme, unless compelling evidence shows that other ways of scoring FHD are more effective at reducing its severity within a selective breeding context.

In dogs, larger breeds are most commonly associated with the highest prevalence of HD. The genetic correlation analyses in our study clearly demonstrate that some of the genes responsible for the larger body type seen in the Maine Coon also increase FHD risk, either because of genetic pleiotropy or inheritance of a linked gene complex. Thus breeding for a large body type carries with it the increased risk of FHD. This finding was further supported when we looked at the average body size of cats within the selective breeding programme against FHD; here as the number of generations of selection against FHD increased, body size decreased. This has serious implications for how the breed standard should be interpreted by breeders and show judges. The Maine Coon breed standard describes the breed as large in type (e.g. “*…the optimum being a large*, *typey cat…the Maine Coon is large framed…medium to large in size*”; mcbfa.org; cfa.org; fifeweb.org). This in itself is not a bad thing; however, there is likely to be temptation for some judges and breeders to favour the larger-than-average cats within the breed, to accentuate one of its defining features. This temptation is hinted at in the same Maine Coon breed standards when they specifically warn against this (“*Males may be larger*, *females are usually smaller*. *Females should not be penalized because of this size difference… Quality should never be sacrificed for size…Type must not be sacrificed for size*”). Our study shows that these warnings need to be heeded, as selecting for a larger-than-average body type is likely to carry with it the unwanted genetic consequences of higher FHD risk. This raises ethical questions about exactly what traits should be promoted in the breed, and the possible trend that these cats are getting larger over time.

Our study is the first to estimate heritability of FHD, examine its genetic correlation with a breed-standard trait and measure response to selective breeding against FHD in a monitored health programme. The data also add considerable weight to our knowledge about the demography of FHD in the Maine Coon^14^ and have significant implications for how we should view the prevalence and management of FHD. Thus, there are associated welfare and ethical issues to consider, not only from a veterinary perspective, but also from the perspective of cat societies and their members. It is important to keep in mind that our focus on the Maine Coon is not just because of its risk profile for FHD, but also because it is the only breed with the data to support such an investigation. Feline hip dysplasia is a significant clinical problem affecting millions of cats worldwide, and it is time it received similar consideration as its canine counterpart.

## Methods

### PawPeds health programme for FHD

The international feline health program against FHD was started by the Maine Coon breeders club “Maine Coon-katten” in Sweden, January 2000, and is currently administered by PawPeds (https://pawpeds.com/healthprogrammes/). This was initiated because of early reports that FHD had a higher prevalence in Maine Coons compared to other breeds^11,25^. The basis for the programme is a standardised score for each hip (ranked from 0–3) based on a ventrodorsal radiographic assessment (Fig. 1). One radiographic specialist is responsible for all evaluations, and scores each hip separately according to four grades: grade 0 = no evidence of FHD, grade 1 = mild dysplasia, grade 2 = moderate dysplasia, and grade 3 = severe dysplasia (for specific grading details see below). The breeding recommendation is to evaluate the hip status of the cat >10 months old but prior to breeding. Cats with grade 2 or grade 3 on either hip are recommended to be excluded from breeding. Cats with grade 1 on either or both hips are not excluded from breeding because of concerns that removing these cats would have unreasonably limited the breeding population; however, the recommendation is that these cats are only mated to cats that have normal hip status in both joints.

### Radiographic assessment of FHD

All radiographs in the PawPeds FHD programme are assessed by a single radiographic expert. Prior to evaluation and grading, the technical quality of radiographs is assessed and only those that conform to strict guidelines are graded (see https://pawpeds.com/healthprogrammes/HDinfoVet.html). Evaluation of the radiographic FHD categories is based on joint conformation (misshapen acetabulum and/or femoral head), fit (loose, limited or uneven contact between joint surfaces) and signs of degenerative joint disease (e.g. bone remodelling and or new bone formation). Specifically, the hip score grades were determined using the following criteria: Hip Score = 0 (normal) - no signs of hip dysplasia and/or degenerative joint disease and the acetabulum covering at least 50% of the femoral head; Hip Score = 1 (mild hip dysplasia) - mild signs of hip dysplasia and/or the acetabulum covering less than 50% of the femoral head but no signs of deforming degenerative joint disease; Hip Score = 2 (moderate hip dysplasia) - moderate signs of hip dysplasia and/or signs of deforming degenerative joint disease; 3 (severe hip dysplasia) - severe signs of hip dysplasia and/or deforming degenerative joint disease (see Fig. 1 for examples). Where hip scores were borderline, these animals were always graded at the lower score: i.e. scores were conservative in these cases.

### Data

From the PawPeds health database, we extracted data for Maine Coons from January 2000 to June 2019 that included 5038 records (female = 3287 & male = 1751) from individuals with information on FHD scores, sex, age and parentage. Of these records, 2156 also had data on the individuals’ body weight (female = 1402 & male = 754) allowing body-weight-related analyses on this subset. We also had access to the Maine Coon pedigree database (https://pawpeds.com) that allowed us to derive information on the genetic relationships between these individuals (for a summary of the pedigree database see Supplementary Table S4).

### Analyses

Our initial approach was to examine the prevalence of different FHD scores based on sex to get a general demographic overview of the data for comparison to^14^ (Table 1). These data were collected during a 20-year period, thus we also wanted to examine whether there was any general trends in the relative proportion of FHD scores during this time that might reflect the influence of the breeding programme. Because the hip-score rankings (0–3) were ordinal, but the proportional odds assumption for an ordered logistic regression was violated (chi-square *P* = 0.01), we used the more general multinomial logistic regression using the ‘mlogit’ package^26^ in R^27^ for this analysis.

Symptoms of FHD are likely related to an interaction between the age and weight of an individual and its genetics. Thus we were interested in how age and weight influenced the phenotypic expression of FHD in male and female cats. Because body mass is age and sex dependent, we converted each individual’s body mass data to a sex- and age-adjusted residual body mass; thus, negative residuals were for individuals smaller than expected, and positive residuals for individuals larger (heavier) than expected for their age (for details see Supplementary Fig. S4). FHD scores displayed a classic Poisson distribution and thus we could use a log-link Poisson regression (truncated to a maximum of 3) to estimate the effect of age and residual body mass on FHD expression (see Supplementary Appendix S1a for full model details). To examine the effect of selection on FHD expression within the PawPeds health programme, for each individual we used information from the pedigree to determine how many generations of their ancestors had been previously assessed for FHD (and thus been selected for within the programme). Thus each individual received a measure of how many generations of selective breeding preceded them, on both their maternal and paternal sides. These were then averaged to give a mean ancestor score that allowed us to estimate changes in the mean FHD score relative to the number of generations of selective breeding. As with the preceding analysis, we used a truncated Poisson regression to estimate this relationship and included age and year to control for age and year effects (see Supplementary Appendix S1b for full model details).

To estimate heritability we needed to statistically partition the phenotypic variance (V_P_) of FHD into its additive genetic (V_A_) and the residual or additional variance components. To do this we fitted a generalised linear mixed-effects model known as an ‘animal model’ in quantitative genetics^28^. The animal model uses information about the genetic relationships between individuals from the pedigree (Supplementary Table S4) to estimate V_A_; from this the heritability (*h*^2^) is then calculated using the formula h^2^ = V_A_/V_P_. Models were implemented using the R package ‘MCMCglmm’^29^ following pedigree checking using the ‘pedantics’ package^30^. These animal models were formulated as ‘threshold’ models for ordinal data, with the unit variance fixed to 1 and individual ID included to account for repeated measures on the same individual (see Supplementary Appendix S1c for full model details). Ordinal threshold models use a (probit) link function to translate between the underlying latent statistical variable (which is continuous) and the observed state of the phenotype (in this case, discrete states from 0–3); thus, *h*^2^ estimates from these models are based on the underlying latent variable rather than the observed state of the phenotype. This means that *h*^2^ estimates from non-gaussian models need to be transformed to account for the variance associated with the link function if *h*^2^ is to be interpreted in the same scale as the observed phenotypic data^31^. Thus for *h*^2^ estimates of threshold models we present them at both the latent scale and observed data scale (after transformation using the ‘QGglmm’ package)^31^.

We then estimated the *h*^2^ of residual body mass and its genetic correlation to the FHD scores by using an extension of the animal model which permits variance to be partitioned between multiple traits^28^. This allowed us to ask whether the phenotypic covariance we observe between residual body mass and FHD scores was due to additive genetic effects (COV_A_). Genetic covariance between traits may occur because they share the same genes or linked gene complexes and is expressed as the genetic correlation (*r*_*G*_) using the formula: *r*_*G*_ = COV_A_/$\sqrt{{V}_{A1}}\times {V}_{A2}$ (see Supplementary Appendix S1d for full model details). As a further confirmation that genetic covariances might exist between body size and FHD, we examined how residual body mass changed relative to the number of generations of selective breeding within the PawPeds programme. Here we expected residual body mass to decline as the number of generations increased, if there was the expected positive genetic correlation between residual body mass and FHD (i.e. the direct selection against FHD would indirectly select against larger body mass; see Supplementary Appendix S1e for full model details).

For the FHD response variable used in these analyses we used each individual’s maximum hip score (e.g. if a cat had a left hip score = 1 and a right hip score = 2, its maximum hip score = 2) because: (1) it seemed reasonable to classify a cat based on its worst HD score, since both hips were not always affected equally (Table 1 & Supplementary Table S2; Fig. S1), (2) this variable has been used in canine HD studies^1,3^, and (3) the genetic correlation between maximum hip score and the left or right hip scores was extremely high (0.996; see Table 2) supporting the idea that it captured the genetic variation we were generally seeking to explain. With the exception of the multinomial modelling of the cross-sectional data, all models were implemented in a Bayesian statistical framework with minimally-informative priors. For the regression models, these were run in JAGS^32^ called from R^27^, and for the quantitative genetic models these were run in MCMCglmm^29^. In all cases models were run until MCMC chains converged and were checked for model fit using posterior predictive checks^33^ (see Supplementary Appendix S1). Results are generally presented as means and the 95% credible intervals from the posterior distributions (i.e. the range where the value of the estimate occurs with a 95% probability) unless otherwise stated.

## Supplementary information

Supplementary information

## Data Availability

All data used in this study are available from PawPeds (https://pawpeds.com/healthprogrammes/): the Maine coon data on FHD were retrieved from the Hip Dysplasia database, and the weight data used to create the growth models were retrieved from the Hypertrophic Cardiomyopathy database.
