# Supplementary material for: Demography, heritability and genetic correlation of feline hip dysplasia and response to selection in a health screening programme
Source: Sci Rep. 2019 Nov 20;9:17164. doi: 10.1038/s41598-019-53904-w (PMC6868272; doi:10.1038/s41598-019-53904-w)
Supplement: Supplementary file 1 — Supplementary information [file 41598_2019_53904_MOESM1_ESM.pdf]

Supporting Supplementary Material for the Paper:

Demography, heritability and genetic correlation of feline hip dysplasia and response to selection in a health screening programme

Matthew Low, Per Eksell, Kjell Högström, Ulrika Olsson, Lars Audell & Åsa Ohlsson

---

Table S1: Additional demographic information from the raw FHD data used in the study

Table S2: Multinomial coefficients for the effect of year on the proportion of individuals in the different FHD categories

Table S3: Regression model coefficients

Table S4: Summary of information contained within the Maine Coon pedigree that was used to estimate the heritability of FHD

Figure S1: Figure showing the relationship between left and right hip scores

Figure S2: Figure showing the raw data for the proportion of cats with moderate-severe FHD from 2000-2019

Figure S3: Elaboration on why cats with unilateral FHD have less severe symptoms than those with bilateral FHD

Figure S4: Figure showing the relationship between age and body weight in Maine Coon cats

Appendix S1a-f: Formal descriptions for the statistical models used in this paper

---

Table S1. Additional demographic information from the raw FHD data used in the study. Here we show the proportion of all animals and sex-specific data for the animals with no evidence of FHD (both hips = 0), those with FHD in both hips (both hips >0) and those animals with FHD only found in one hip (one hip = 0, one hip > 0). We also show the breakdown of the different FHD scores depending on whether the cat has bilateral or unilateral FHD.

Condition	Percentage
All animals (n = 5038)	
Both hips = 0	62.6%
Both hips > 0	23.6%
One hip = 0, One hip > 0	13.8%
if both hips FHD	score 1 = 54.0%, 2 = 33.2%, 3 = 12.8%
if only one hip FHD	score 1 = 85.1%, 2 = 14.3%, 3 = 0.6%
Females (n = 3287)	
Both hips = 0	62.9%
Both hips > 0	23.4%
One hip = 0, One hip > 0	13.7%
if both hips FHD	score 1 = 55.2%, 2 = 33.0%, 3 = 11.8%
if only one hip FHD	score 1 = 85.7%, 2= 13.4%, 3 = 0.9%
Males (n = 1751)	
Both hips = 0	62.1%
Both hips > 0	24.0%
One hip = 0, One hip > 0	13.9%
if both hips FHD	score 1 = 51.4%, 2 = 33.6%, 3= 14.8%
if only one hip FHD	score 1 = 84.1%, 2 = 15.9%, 3 = 0%

Table S2. Multinomial coefficients for the effect of year on the proportion of individuals in the different FHD categories (0 = no radiographic signs of HD, to 3 = severe radiographic signs of HD). Here the coefficients are not directly interpretable as their derived estimates need to be inverse logit transformed and scaled so the probabilities of all levels sum to one (see Fig. 2).

Coefficients	Estimate ± SE	z-statistic	p-value
Males			
intercept 1	-1.88 ± 0.18	10.4	<0.0001
intercept 2	-3.29 ± 0.25	13.1	<0.0001
intercept 3	-3.96 ± 0.38	10.4	<0.0001
year effect 1	0.029 ± 0.012	2.29	0.021
year effect 2	0.089 ± 0.016	5.46	<0.0001
year effect 3	0.079 ± 0.025	2.56	0.010
Females			
intercept 1	-2.17 ± 0.13	16.7	<0.0001
intercept 2	-2.82 ± 0.18	15.6	<0.0001
intercept 3	-4.22 ± 0.29	14.5	<0.0001
year effect 1	0.051 ± 0.009	5.62	<0.0001
year effect 2	0.051 ± 0.012	4.34	<0.0001
year effect 3	0.073 ± 0.019	3.79	0.0001

Table S3. Model coefficients (means ± SD from the posterior distribution) from the models detailed in Figs 3 & 4; Appendices S1a, b & e: (model 1) max hip score ~ age + residual body mass; (model 2) max hip score ~ generations + age + year; (model 3) residual mass ~ generations + age. Coefficients for models 1 & 2 are at the log scale (Poisson), while model 3 is at the observation scale (Gaussian). In all models, age is mean centred and standardised to 1 standard deviation, year is number of years since the start of the programme (beginning at 1).

Coefficients	All	Males	Females
model 1 (log)			
max hip score			
intercept		-1.13 ± 0.10	-1.26 ± 0.08
residual mass		0.28 ± 0.05	0.27 ± 0.06
age (std)		0.12 ± 0.05	0.11 ± 0.04
model 2 (log)			
max hip score			
intercept		-0.14 ± 0.08	-0.74 ± 0.03
generations		-0.139 ± 0.028	-0.145 ± 0.021
age		0.07 ± 0.03	0.05 ± 0.02
year		0.05 ± 0.01	0.04 ± 0.01
model 3			
residual mass			
intercept	-0.086 ± 0.042		
generations	-0.033 ± 0.016		
age (std)	-0.027 ± 0.022		

Table S4. Summary of information contained within the Maine Coon pedigree that was used to estimate the heritability of FHD by statistically partitioning the phenotypic variance of FHD into its additive genetic and the residual variance components using a quantitative genetic 'animal model'. Summary table generated using the 'pedantics' package[30] in R.

Pedigree parameters	Data
records	8902
maternities	5007
paternities	5011
full sibs	1551
maternal sibs	3444
maternal half sibs	1893
paternal sibs	7189
paternal half sibs	5638
maternal grandmothers	2511
maternal grandfathers	2512
paternal grandmothers	2265
paternal grandfathers	2265
maximum pedigree depth	14
founders	3886
mean maternal sibsip size	1.535889571
mean paternal sibsip size	1.954368175
non-zero F	236
F > 0.125	2
mean pairwise relatedness	0.000645316
pairwise relatedness>=0.125	0.001759563
pairwise relatedness>=0.25	0.000821185
pairwise relatedness>=0.5	0.000292163

Figure S1. Relationship between left and right hip scores for the 5038 individual FHD assessments used in our study (summarised in Table 1 & S2). Females are shown in red and males in blue. Noise was added to each point so it could be visualised in the plot.

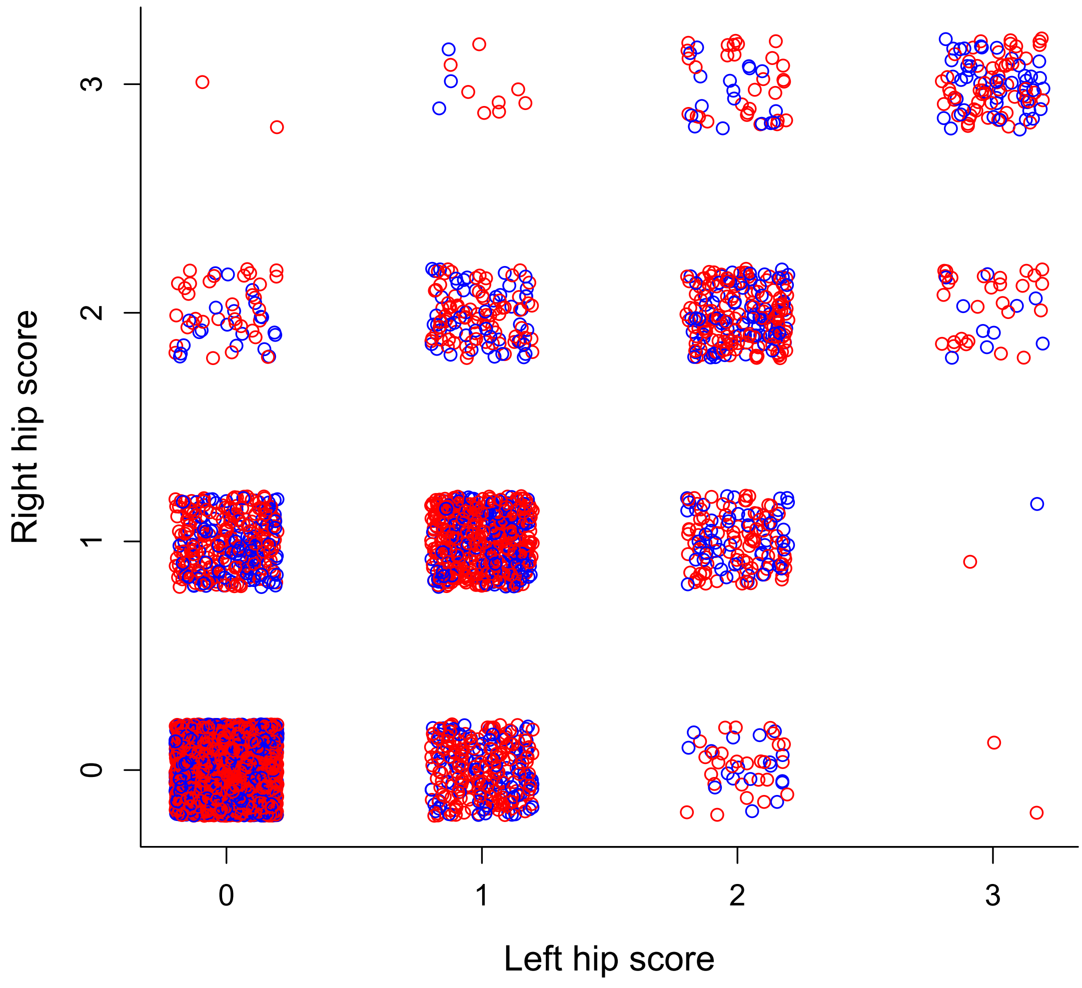

Figure S2: Raw data showing the proportion of cats assessed in the PawPeds health screening programme with moderate (hip score = 2, left panel) and severe hip dysplasia (hip score = 3, right panel) relative to the year of assessment.

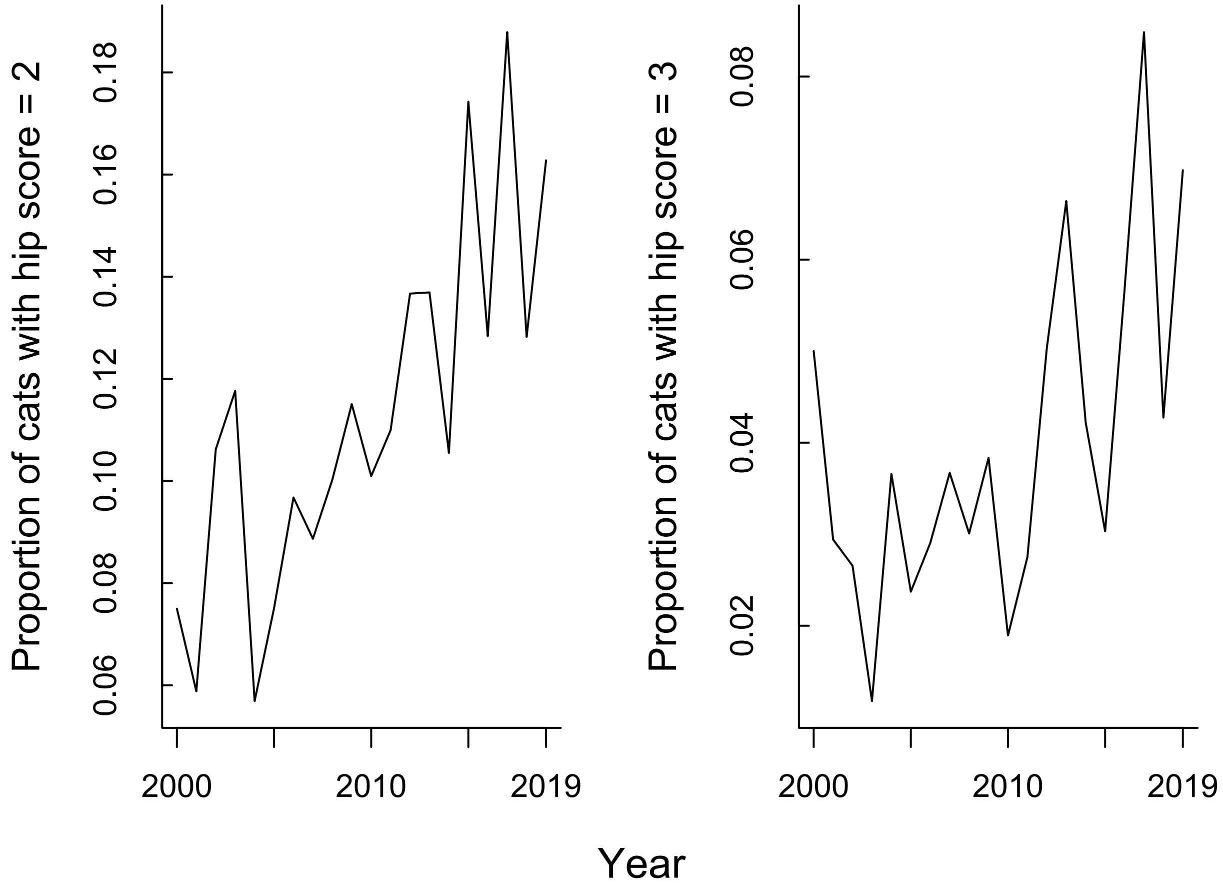

Figure S3: Using sampling from a Poisson distribution (truncated between 0 and 3) to demonstrate why laterality in FHD is related to severity. 100 random samples were taken from a truncated Poisson distribution for a mean (lambda) = 1 (mild FHD; left) and for a mean (lambda) = 3 (severe FHD; right). Cats with a mild genetic predisposition (left panel, mean = 1) have a 37% probability of having a hip score of zero by chance. Cats with a high genetic predisposition and severity (right panel, mean = 3) have only a 7% probability of having a hip score of zero by chance. Thus cats with mild symptoms are more likely to be unilateral in their clinical presentation simply because of sampling variation than cats with severe FHD.

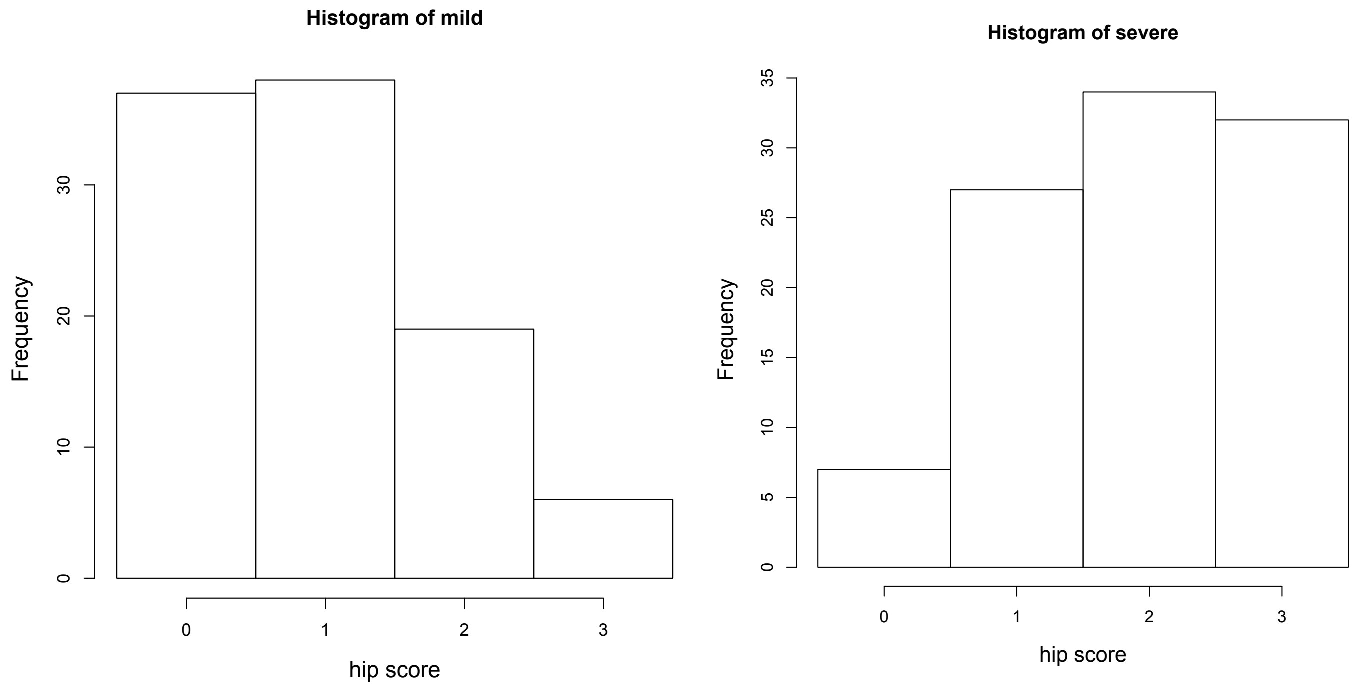

Figure S4. Raw body mass data (grey points) and predicted mean body mass for male and female Maine Coons, relative to their age (days). Predictions were generated using data taken from the PawPeds Maine Coon database for Hypertrophic Cardiomyopathy because it had a large amount of sex-specific data for individuals with corresponding weight and age data (5675 females and 3275 males with age ranges from 207-5151 days [females] and 204-5325 days [males]). Using these data we fitted sex-specific models based on a Michaelis-Menton growth curve function [body mass = A * age / (B + age)] (see Appendix S1f for full model details). Residual body mass for the individuals in our study were then derived from the mean predictions generated by these models, as the deviation from the expected age-dependent size. Thus negative residuals were for individuals smaller than the age-predicted mean; positive residuals for individuals larger (heavier) than expected for their age.

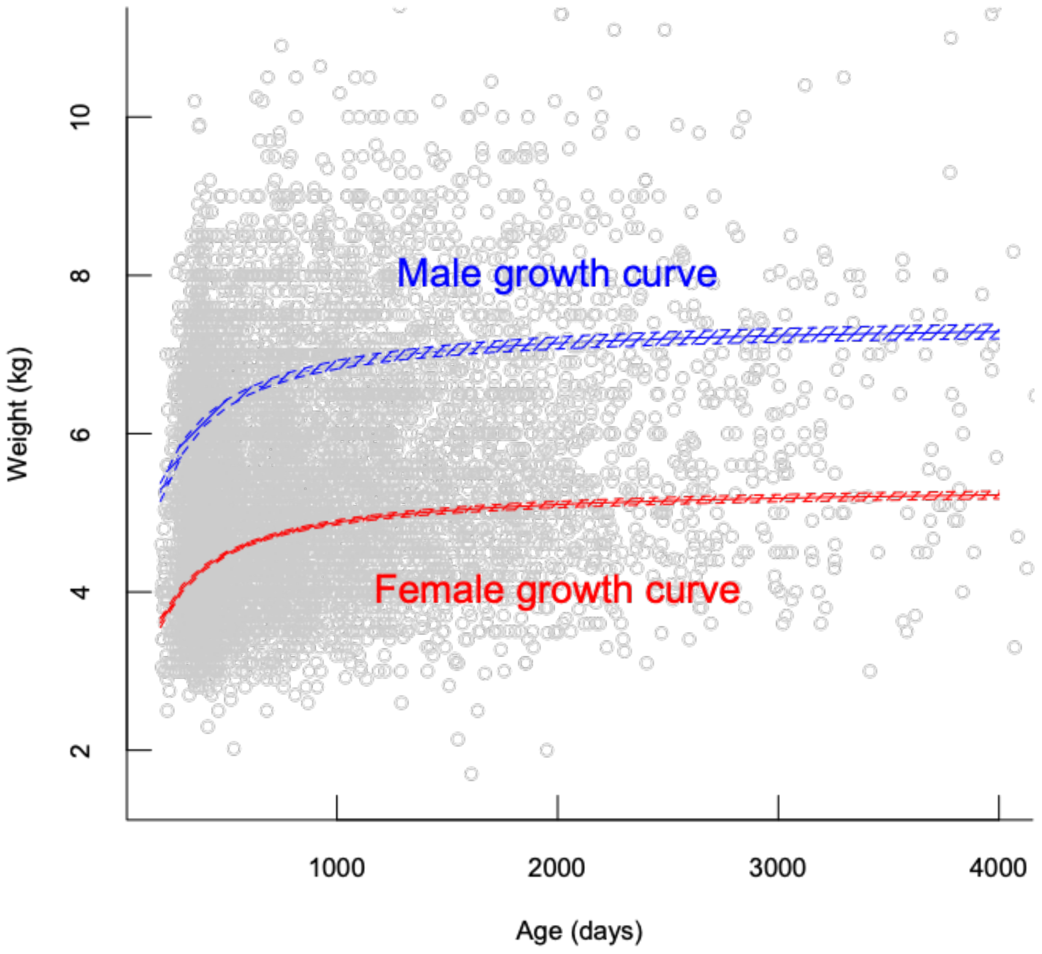

Appendix S1a. Model formulation for the regression examining the relationship between age, residual body mass to FHD max score (Fig. 3). Here the poisson distribution has been truncated to between 0-3. Data used were the maximum hip score for each individual (from 0-3), their age (in days) and their residual body mass (as calculated from the model described in Fig. S1). Models were run for 20,000 iterations after 10,000 burn-in and chains were visually inspected for stability. Posterior predictive checks show that the model is well fitted for both the mean and the variance (Bayesian P = 0.54 and 0.85 respectively; these should be between 0.1 & 0.9). Estimates of the model coefficients are listed in Table S4.

$$\text{max hip score}_i \sim Poisson(\lambda_i)\ T(0,3)$$

$$\log(\lambda_i) = \alpha + \beta_1 * age_i + \beta_2 * residualBM_i$$

$$\alpha \sim Normal(0,100)$$

$$\beta_1 \sim Normal(0,100)$$

$$\beta_2 \sim Normal(0,100)$$

Appendix S1b. Model formulation for the regression examining how expected maximum FHD scores change as the number of generations of selective breeding in the PawPeds health programme increases (Fig. 4a). Here the poisson distribution has been truncated to between 0-3. Data used were the maximum hip score for each individual (from 0-3), the average number of parental generations that preceded each individual in the programme, their age (in days) and year since the programme began. Models were run for 20,000 iterations after 10,000 burn-in and chains were visually inspected for stability. Posterior predictive checks show that the model is well fitted for both the mean and the variance (Bayesian P = 0.51 and 0.79 respectively; these should be between 0.1 & 0.9). Estimates of the model coefficients are listed in Table S4.

$$\text{max hip score}_i \sim Poisson(\lambda_i)\ T(0,3)$$

$$\log(\lambda_i) = \alpha + \beta_1 * age_i + \beta_2 * generations_i + \beta_3 year_i$$

$$\alpha \sim Normal(0,100)$$

$$\beta_1 \sim Normal(0,100)$$

$$\beta_2 \sim Normal(0,100)$$

$$\beta_3 \sim Normal(0,100)$$

Appendix S1c. Models and their priors for calculating the heritability of max FHD score (a) and residual body mass (b). This is the code for implementing the models and extracting their outputs using the 'MCMCglmm' package (Hadfield 2010) and for converting the heritability outputs to the observed data scale using 'QGglmm' package (de Villemereuil 2018) in R (R Core Team 2018).

(a)

```
prior <- list(R=list(V = 1 , fix=1), G = list(G1=list(V=1,nu=1,
 alpha.mu=0, alpha.V=1000),G2=list(V=1, nu=1, alpha.mu=0, alpha.V=1000)))

model <- MCMCglmm(max ~ 1, random = ~animal + ID, family = "threshold",
 prior = prior, pedigree = mcmc.ped, data = data, nitt = 500000, burnin =
 100000, thin = 100)

#latent scale h2
herit <- model$VCV[, "animal"]/(model$VCV[, "animal"] + model$VCV[,
 "units"])
mean(herit)
HPDinterval(herit)

#observed data scale
mu<-model$Sol[, "(Intercept)"]
va<-model$VCV[, "animal"]
vp<-rowSums(model$VCV)

store<-numeric(length(mu))
for(i in 1:length(mu)){
hold<-QGparams(mu=mu[i], var.a=va[i], var.p=vp[i], model="threshold",
 verbose=F)
store[i]<-as.numeric(hold[4])
}
mean(as.mcmc(store))
HPDinterval(as.mcmc(store))
```

(b)

```
prior <-list(R = list(V=1, nu=0.002), G = list(G1 = list(V=1,nu=0.002)))
model <- MCMCglmm(residBM ~ 1, random = ~animal, family = "gaussian",
 prior = prior, pedigree = mcmc.ped, data = data, nitt = 50000, burnin =
 10000, thin = 10)

herit <- model$VCV[, "animal"]/(model$VCV[, "animal"] + model$VCV[,
 "units"])
mean(herit)
HPDinterval(herit)
```

Appendix S1d. Model and priors for calculating the genetic correlation between max FHD and residual body mass using a bivariate model structure in the 'MCMCglmm' package (Hadfield 2010) in R.

```
prior<-list(R=list(V=diag(2), nu=1.002, fix=2),G=list(G1=list(V=diag(2)/
 2,nu=2, alpha.mu=c(0,0), alpha.V=diag(c(1000,100)))))


model <- MCMCglmm(cbind(resid,max) ~ trait-1, random = ~us(trait):animal
, family = c("gaussian", "threshold"), prior = prior, pedigree =
 mcmc.ped, data = data, rcov=~us(trait):units, nitt = 500000, burnin =
 100000, thin = 200)

genetic.correlation<-model$VCV[, "traitmax:traitresid.animal" ]/
 sqrt(model$VCV[, "traitresid:traitresid.animal" ]*model$VCV[,
 "traitmax:traitmax.animal" ])
mean(genetic.correlation)
HPDinterval(genetic.correlation)
```

Appendix S1e. Model formulation for the regression examining how residual body mass changes as the number of generations of selective breeding in the PawPeds health programme increases (Fig. 4b). Data used were the residual body mass scores (kg), the average number of parental generations that preceded each individual in the programme, and their age (in days). Models were run for 20,000 iterations after 10,000 burn-in and chains were visually inspected for stability. Posterior predictive checks show that the model was well fitted for both the mean and the variance (Bayesian P = 0.51 and 0.48 respectively; these should be between 0.1 & 0.9). Estimates of the model coefficients are listed in Table S4.

$$\text{residual body mass}_i \sim Normal(\mu_i, \sigma)$$
$$\mu_i = \alpha + \beta_1 * age_i + \beta_2 * generations_i$$

$$\alpha \sim Normal(0,100)$$
$$\beta_1 \sim Normal(0,100)$$
$$\beta_2 \sim Normal(0,100)$$
$$\sigma \sim Uniform(0,50)$$

Appendix S1f. Michaelis-Menton growth model for relating body mass to age. Mean predictions from sex-specific models were used to determine the age-specific residual body mass data used in the analyses in the paper. Estimates for females were (A = 5.35, B = 96.54) for males (A = 7.43, B = 82.88) and age (days).

$$\text{body mass}_i \sim Normal(\mu_i, \sigma)$$
$$\mu_i = A * age_i / (B + age_i)$$

$$A \sim Gamma(0.001, 0.001)$$
$$B \sim Normal(0, 100)$$
$$\sigma \sim Uniform(0, 50)$$
